# Tunneling Nanotubes: A New Target for Nanomedicine?

**DOI:** 10.3390/ijms23042237

**Published:** 2022-02-17

**Authors:** Ilaria Ottonelli, Riccardo Caraffi, Giovanni Tosi, Maria Angela Vandelli, Jason Thomas Duskey, Barbara Ruozi

**Affiliations:** 1 Clinical and Experimental Medicine PhD Program, University of Modena and Reggio Emilia, 41125 Modena, Italy; ilaria.ottonelli@unimore.it; 2 Nanotech Lab, Te.Far.T.I., Department of Life Sciences, University of Modena and Reggio Emilia, 41125 Modena, Italy; riccardo.caraffi@unimore.it (R.C.); gtosi@unimore.it (G.T.); mariaangela.vandelli@unimore.it (M.A.V.); ruozi.barbara@unimore.it (B.R.)

**Keywords:** nanomedicine, tunneling nanotubes, nanoparticles, drug exchange, therapeutic efficiency, targeted therapy

## Abstract

Tunneling nanotubes (TNTs), discovered in 2004, are thin, long protrusions between cells utilized for intercellular transfer and communication. These newly discovered structures have been demonstrated to play a crucial role in homeostasis, but also in the spreading of diseases, infections, and metastases. Gaining much interest in the medical research field, TNTs have been shown to transport nanomedicines (NMeds) between cells. NMeds have been studied thanks to their advantageous features in terms of reduced toxicity of drugs, enhanced solubility, protection of the payload, prolonged release, and more interestingly, cell-targeted delivery. Nevertheless, their transfer between cells via TNTs makes their true fate unknown. If better understood, TNTs could help control NMed delivery. In fact, TNTs can represent the possibility both to improve the biodistribution of NMeds throughout a diseased tissue by increasing their formation, or to minimize their formation to block the transfer of dangerous material. To date, few studies have investigated the interaction between NMeds and TNTs. In this work, we will explain what TNTs are and how they form and then review what has been published regarding their potential use in nanomedicine research. We will highlight possible future approaches to better exploit TNT intercellular communication in the field of nanomedicine.

## 1. Tunneling Nanotubes 

### 1.1. What Are Tunneling Nanotubes?

Tunneling nanotubes (TNTs), first described in the literature in 2004 by Rustom et al. [1], have gained growing interest from the scientific community. They are described as long and thin protrusions of the cytoskeleton and plasma membrane which connect two different cells, extending distances even up to several µm [2,3]. The composition of these bridges is simple, as they are normally composed of actin and tubulin filaments surrounded by plasma membrane; however, the presence of tubulin has also been reported as variable, leading to the classification of two different types of TNTs: (1) “thin” TNTs, composed of only actin, which are usually more delicate and transient, and (2) “thick” TNTs, with both actin and tubulin, which are often associated with a more stable structure [4]. TNTs have peculiar features which distinguish them from other cell protrusions. TNTs differ from filopodia, cilia, or cytonemes, both in their structure and function: TNTs are very thin filaments which do not adhere to the substratum, but more importantly, they present open endings in the plasma membrane of the two cells they are connecting. Moreover, these open endings allow for the direct exchange of virtually any kind of cargo from one cytoplasm to another: they allow for the transport of not only of ions and neurotransmitters, but also whole organelles, proteins, and genetic material [2,5]. A more detailed explanation is included in a review by Pinto et al., with a comprehensive table explaining the differences and relevant citations [5]. In physiological conditions, these bridges have been demonstrated to be essential not only for the embryonic development [6,7,8,9] of vertebrates, but also in their adult form to maintain a healthy status of their tissues. For example, TNTs have been demonstrated to be critical not only for preserving the differentiation potential of mesenchymal cells [10], but also for repairing damages in other neighboring cells by exchanging intact organelles [11,12,13]. Moreover, TNTs are involved in the exchange of electrical and chemical signaling in different tissues, such as in the eye [14,15]. It was reported that an insufficient communication via TNTs in the trabecular meshwork is linked to an increase in intraocular pressure, and consequently, an increased risk of glaucoma [16]; at the same time, TNTs are involved in the transmission of calcium ions in the retina, in determining good health, and in correctly firing retinal photoreceptors [17,18].

Notwithstanding their essential role in physiological conditions, TNTs are better known for to their involvement in pathological processes. As reported in several recent review works, TNTs play a key role in the spreading of several disease states, such as neurodegeneration, infections, and cancer. In the case of neurodegenerative diseases, the literature shows that cells can use TNTs to transport prions, misfolded huntingtin, Tau protein, α-synuclein, and β-amyloid, promoting protein misfolding in other cells [19]. This additive effect increases the risk of developing Huntington’s [20,21], Parkinson’s [22,23], and Alzheimer’s diseases [24,25,26]. Another field in which TNTs play a pivotal role is cancer. The exchange of misfolded proteins and damaged genetic material through TNTs in cancers is considered one of the major phenomena that contribute to the transformation of healthy cells into tumoral cells and increases in metastasis formation [5,27]. While TNTs have been linked to communication and spreading in several types of cancer [28], such as prostate [29], bladder [30,31,32], pancreatic [33], and breast cancer [34], as well as different types of leukemia [35,36,37]. Glioblastoma multiforme (GBM) is by far the most studied for the consequences of TNT activity [4,38]. GBM is one of the most aggressive, invasive, and fatal brain cancers [39], with a survival of less than 15 months after diagnosis [40,41]. In fact, the fast growth and invasiveness of GBM have been linked to TNT-mediated communication between GBM cells towards and surrounding healthy astrocytes [42,43]. This could be linked to the reason for why the vast majority of studies about TNTs are performed using GBM cells. The high rates of TNT formation in these cells makes them an optimal in vitro model to study their mechanics and dynamics [44].

The natural exchange of different materials from one cell to another is an evolutionary defensive strategy to reduce the risk of cell death: on one hand, a healthy cell could share its organelles with a diseased one to promote damage repair [45,46] or improve cell respiration by the transfer of mitochondria in case of hypoxia [47]; on the other hand, this can also be used by cells in an attempt to dilute stressful inputs, leading to an increased number of stressed cells but lower stress levels. However, these mechanisms are also exploited and enhanced by numerous viruses, such as HIV [48], herpesviruses [49,50], influenza viruses [51,52], and more recently, SARS-CoV-2 [51,53,54]. After viral replication, the infected cell will be in an inflammatory state that causes the formation of a larger number of TNTs, in order to reduce the stress on the primary cell. With these mechanisms, viruses exploit this highway to increase the number of infected cells while also reducing the risk of recognition by the immune system outside the plasma membrane [55]. The same pattern was observed in vitro after the administration of cytotoxic drugs, where the cells were demonstrated to promote the efflux of toxic compounds and share it with neighboring cells [56], often with a linear correlation between the amount of cytotoxic drug administered and the number of TNTs formed by cells [33]. While this is a protective reaction of the cell to dilute the toxin, it could also be a pitfall leading to lethal levels of the drugs in all surrounding cells. Another consequence of this is that TNTs are considered one of the major mechanisms involved in the onset of chemoresistance [57], as the simultaneous transport via the TNTs of drugs, P-glycoproteins, and microRNAs all contribute to multidrug resistance [58,59,60]. 

### 1.2. Exogenous Modulation of Tunneling Nanotubes

It is clear that in order to take advantage of this intercellular cross-talk, a deeper study of the physiological and pathological role of TNTs in different tissues is needed. As previously described, while the inhibition of TNTs could help reduce the spreading of tumors and diseases, in other cases, promoting their formation might improve the localized cellular distribution of therapeutic molecules. To date, inhibitors of the formation of TNTs mostly block the mobility of the whole cytoskeleton [61,62]. For example, latrunculin B, the most-used compound to affect TNTs, is an inhibitor of actin polymerization that affects the whole cell. Similarly, other small molecules such as metformin and everolimus are able to reduce the number of TNTs formed by cells due to their role as inhibitors on the mTOR pathway [63,64]. Nevertheless, most of these compounds are considered toxic for cells because their effect is not limited to reducing the formation of TNTs, but they affect the mobility of the whole cytoskeleton. This non-specific inhibition of physiological processes such as cell migration and mobility disrupts normal cell function and growth, leading to devastating effects. Interestingly, tolytoxin was reported to have a selective effect of inhibiting the formation of TNTs without any general effects on the cytoskeleton, thus representing a valuable tool for limiting the intercellular transport via TNTs [65].

On the contrary, the induction of TNTs seems to be easier to achieve. It has been abundantly demonstrated in the literature that the formation rate of TNTs in vitro can be increased by several inputs linked to the culture protocol. This includes variables such as low levels of oxygen or a high presence of CO_2_, acidic pH, serum starvation, or low glucose concentrations [5,29,42,45]. All these conditions represent situations of cellular stress in which cells tend to connect in order to improve their survival, as previously described. Another widely used technique to induce the formation of TNTs in vitro is also to transfect cells with proteins involved in cytoskeletal mobility and cell adhesion. The administration of mSEC [66,67], which triggers the formation of TNTs due to the higher dynamicity of the cytoskeleton, is a primary example. Most drugs used and tested in cell cultures have been also linked to an increase in the connections between cells due to their stressful effect, especially considering anticancer drugs and antibiotics [68,69,70]. 

This has raised the question of whether the same effect is seen by the administration of nanomedicines (NMeds). NMeds, as drug delivery systems, have been studied for more than 30 years, but when administered to cells, they represent a source of stress and could increase the number of TNTs, facilitating the spreading of the loaded drug in the tissue. When designing a NMed-based therapeutic approach, it is crucial to take into account this piece of information: whether it is necessary to reduce or trigger TNT formation. Research in this direction has the potential to change the way we design therapeutic approaches but could represent a great step forward in improving the efficacy and specificity of NMed treatments.

## 2. Nanomedicine

**Figure 1:**
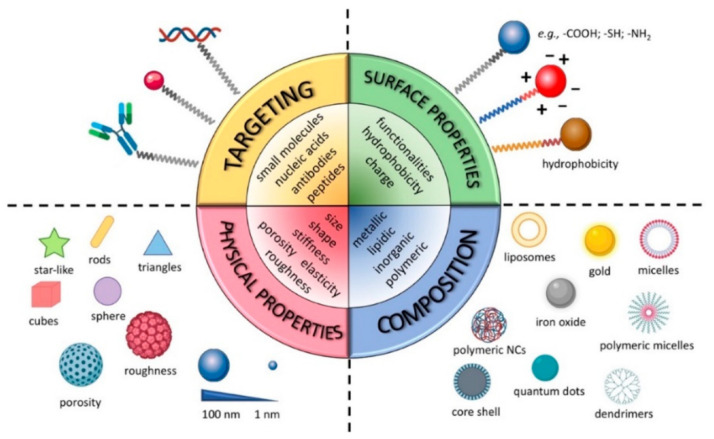
Graphical representation of NMeds’ customization options and advantages. Reproduced with permission from Salvioni et al. [71] (Cancers; published by MDPI; 2019).

NMeds are one of the most investigated tools in drug delivery due to their numerous advantages over traditional pharmaceuticals [71,72,73,74,75,76,77]. NMeds are defined as nanometric-sized delivery systems with a vast range of types that, depending on their specific characteristics, can be optimized to encapsulate, protect, and specifically deliver virtually any kind of therapeutic agent. In particular, the literature results of the last 20 years demonstrate NMeds intelligently designed to (1) improve the solubility of poorly soluble drugs [78,79], (2) stabilize and protect sensitive molecules such as proteins [80,81,82,83], peptides [84,85,86], and genetic material [87,88] from degradation, (3) promote their accumulation into target cells or tissues [89,90], and thereby (4) reduce drug toxicity outside the targeted tissue [91,92], and (5) prolong and/or control the release of the drug over time (Figure 1) [93,94,95,96]. All these properties together make NMeds perfect candidates for the treatment of a plethora of pathologies, especially those considered difficult to treat or that affect difficult-to-reach organs, including neurodegenerative disorders, such as Alzheimer’s [97], Parkinson’s [98], or Huntington’s [99], different types of cancer [100], e.g., breast cancer [101], leukemia [102], or GBM, and numerous other diseases that require penetration of the blood-brain barrier (BBB) [103]. 

The main feature that allows these NMeds to be so widely applied to these pathologies is the possibility of engineering their surface with ligands, such as small molecules [104,105], peptides [77,106,107,108], antibodies [109,110,111], aptamers [112,113], etc., which specifically react with the cell surface to improve localized accumulation at the target site. Targeted delivery can also be achieved by modifying the surface with coating layers [114,115,116,117,118], or environmentally sensitive moieties that react to differences such as pH, ROS, temperature, light, enzymes, etc., in order to promote controlled release only in the relevant microenvironment, often created by a pathological change [119,120,121,122,123,124,125,126,127]. These different ligands have been developed and improved in the last decade to increase their specificity and thereby enhance the ability of NMeds to cross barriers (i.e., BBB or blood–retinal barrier) and/or the accumulation of NMeds only in the target cells [128,129]. Notwithstanding the great advancement in targeting specificity, TNTs are currently under investigation for their potential role in diminishing this targeting effect due to intercellular transport by exchanging NMeds from a correctly targeted cell towards a neighboring off-target one. Remarkably, despite the impact it could have, the topic has been poorly addressed. Here, we review the work that has been completed to demonstrate the interaction between TNTs and NMeds.

## 3. Nanomedicine and TNTs

### 3.1. Inorganic NMeds

The first evidence in 2010 that NMeds travel along TNTs was reported by He et al., and involved the transfer of inorganic nanoparticles [130]. Here, they visualized quantum dots (QD) of CdSe/ZnS being transported along “newly discovered nanotubular structures” formed between rat cardiac myoblast cells. In fact, this study pioneered the idea that NMeds could be transported via TNTs inside membrane vesicles, and that the exchange could be bidirectional, which would afterwards be confirmed by successive publications [131,132]. Similarly, in the following year, Mi et al., reported the intercellular transfer of CdTe QD along TNTs in human hepatocellular carcinoma cells [133]. Here, the authors further distinguished that the transfer of these QD could be unidirectional or bidirectional, depending on the composition of the TNT. It is important to clarify that this bidirectionality was possible only in the presence of tubulin, meaning, thus, in the more stable “thick” TNTs [25]. A more in-depth analysis of the mechanism underlying the transport of these QD showed that they were not transported as single particles, but instead traveled inside lysosomes as aggregates. Although this was not specifically investigated in the study by He et al., it is safe to hypothesize that the QD were transported within lysosomes also in those cultures. Another interesting work was published by Domhan et al. regarding the trafficking of QD, in which the authors demonstrated the transport of two different QD-based fluorophores via TNTs among primary cultures of human tubular epithelial cells [134]. Remarkably, the TNTs were demonstrated to play a key role in the exchange of QD, as modulating their number with exogenous factors such as stress, and the administration of latrunculin B or zeocin, resulted in different rates of NMeds exchange. In fact, these data were the first evidence of the possibility to directly impact the transport of NMeds through TNTs by influencing their formation with external stimuli, even if the precise mechanism has not been characterized. Another important aspect that can be highlighted by these reported examples is that the trafficking of QDs, and of NMeds in general, is not limited to immortalized cells but is also present in primary cultures. This can also be extended further to 3D in vitro models such as organoids and tissues, which will be discussed in later sections. Further confirmation of this effect was reported by Rehberg et al., who detected and tracked the transportation of QD along TNTs in vivo in the cremaster muscle of mice, especially between tissue macrophages [135]. This represents one of the few reports of TNTs in vivo to date.

All the previously cited articles demonstrated the ability of TNTs to transfer NMeds between cells in a monoculture. Nevertheless, an important factor that was ignored in the previously reported works is that TNTs can create connections not only between cells of the same type (homotypical transfer), but also between cells of different types (heterotypical). Interestingly, NMeds can be shared with other cells by both types of transport. This effect was specifically noted in a study by Epperla et al. [136]. In this study, the authors used fluorescent nanodiamonds (FNDs) in both human embryonic kidney cells and neuroblastoma cells. First, it was evidenced that both these cell types were able to form homotypical TNTs when separately cultured. The only difference arose from the thickness and composition of the TNTs naturally formed by each specific cell type. In particular, neuroblastoma cells mainly formed “thin” actin-based TNTs, while HEK cells predominantly formed “thick” TNTs containing both actin and tubulin. In both cell types, the FNDs were exchanged in single cultures, but more remarkably, the authors also documented heterotypical exchange of FNDs between the cells in co-cultures. The authors reported that FNDs spread from predosed HEK cells to neuroblastoma cells when added in the culture. Quantification reported that approximately 10% of the neuroblastoma cells tested positive for FNDs due to the transposition between cells by TNTs. 

**Figure 2:**
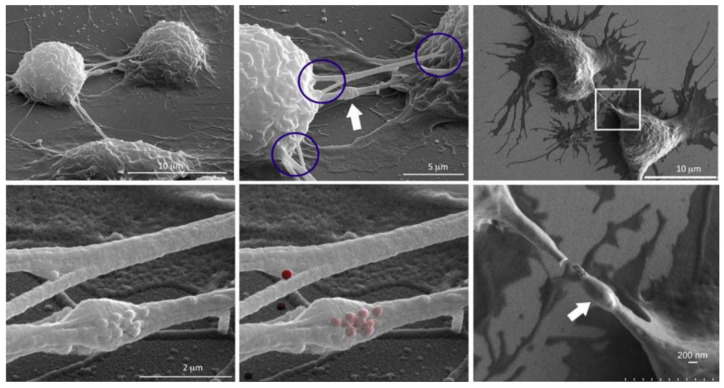
SEM images showing TNTs between macrophages emphasizing disparate sites of connectivity (circled) and the presence of a gondola (white arrow). NMeds are pseudo-colored red in the lower central image. Reproduced with permission from Franco et al. [137] (Cancers; published by MDPI; 2020).

To further investigate heterotypical exchange of NMeds, an interesting TNT study by Franco et al. takes precedence [137]. The authors dosed mesoporous silica nanoparticles into mice macrophages. Results suggested that the presence of these fluorescent NMeds along TNTs were localized in the so-called “gondola” structures, indicating the node where NMeds accumulated during transportation (Figure 2). This study led to a number of reported peculiarities regarding the formation of TNTs and their ability to transport NMeds. First, researchers demonstrated the formation of TNTs between murine macrophages and HeLa cells, indicating that these structures can be formed even between murine and human cells. More importantly, the transfer of NMeds via TNTs was successfully modulated by exogenous factors. In particular, by adding cell stress by serum starvation, the trafficking of NMeds between the two cell lines increased significantly. On the contrary, hyperthermia reduced TNT formation and, consequently, NMed transfer. These results lead to two important points. On one hand, the possible transfer of NMeds to very different cell types calls for a deeper investigation on the dynamics and occurrence: it is crucial to determine the incidence, extent, and direction of the transportation of drug-loaded NMeds. On the other hand, these data were a first step towards controlling TNT formation to modulate NMed delivery. This response to hyperthermia could be critical to the formation of TNTs in other cell types. The idea of controlling TNTs in this simple way could help control NMed delivery to improve therapeutic efficacy and reduce toxicity of the loaded drug in a plethora of diseases, for which optimized NMeds are already produced [83,86,99]. The control over the fate of NMeds is necessary to increase pharmaceutical effects over off-target toxicity. This duality is an important part of TNT research that, until this point, has been poorly addressed and calls for more in-depth studies.

### 3.2. Organic NMeds

While inorganic NMeds are generally easier to produce and characterize, they are less frequently used in therapeutic approaches due to their low biodegradability and the fact that they accumulate unfavorably in the liver and kidney, leading to off-target toxicity [138]. Organic NMeds, on the other hand, are generally more biocompatible, highly versatile, and easy to functionalize on the surface to obtain targeted delivery. For these reasons, polymeric and lipidic NMeds are generally preferred as promising tools for specific targeted delivery. Nevertheless, they also have been demonstrated to undergo intercellular trafficking via TNTs, thus representing a huge limitation to their efficacy. Here, we gathered the works that have analyzed the interaction between polymeric or lipidic NMeds and the formation rate of TNTs.

#### 3.2.1. Polymeric NMeds

Polymeric NMeds have been leading the research field in recent years for their ability to encapsulate both hydrophilic and hydrophobic compounds, their stability, and their high potential in terms of scalability, ease of production, targeting ability, and low material cost. Notwithstanding their advantages, polymeric NMeds are transported along TNTs, thus implying the possibility of uncontrolled biodistribution. Ingle et al., recently reported the trafficking of polyplexes along TNTs in cultured HeLa cells [139]. In particular, they followed the transposition of fluorescently labelled Glycofect/DNA polyplexes in membranous bridges in vitro, showing the transport of these NMeds in vesicles along TNTs. Evidence of this kind of transport leads to the hypothesis of exploiting TNTs in diseased tissues, such as tumors, as highways to increase the biodistribution of therapeutics such as RNAs, enhancing currently used approaches to improve treatments against diseases such as cancer. Unfortunately, this study reported only preliminary results, and in-depth studies on, for example, whether the administration of polyplexes has an impact on the number of TNTs that cells form, as well as studies to compare their formation in tumoral and healthy cells, are still lacking. 

Interesting results were also reported by Sáenz-de-Santa-María and coworkers [140]. In this study, the authors mainly focused on the biological mechanisms underlying the formation of TNTs in cultured squamous cells carcinoma cells. To this end, they monitored the transport of inert methacrylate NMeds along TNTs in cultured cells. They were able to inhibit the formation of TNTs using two different agents, namely, the FAK inhibitors FRNK and PF-562271, the latter being currently investigated for its anticancer activity [141]. Unfortunately, no comparison of the exchange rate of NMeds after modulation of TNTs was performed, which would be pivotal information. Nevertheless, the authors demonstrated the formation of TNTs in tumor spheroid models, making a first pass towards a model that more closely represents the physiological conditions of in vivo experiments. In fact, these results represent a steppingstone to a novel therapeutic approach against cancer, but the correlation between TNTs and NMeds should be further investigated in particular, since it is possible to observe them in a complex 3D model such as a spheroid. This additional information represents the next critical step to assess the possibility for researchers to exploit TNT for the improved transfer and therapeutic effect of NMeds.

Another crucial parameter that is rarely taken into account when performing this type of study is the surface modifications of the NMeds. TNTs represent a major issue in the field of targeted delivery, and therefore, it is crucial to understand how targeted NMeds interact with these structures and how they change the targeting capacity to influence the final localization of the NMed. In 2014, Tosi et al., demonstrated the transfer of BBB-targeted NMeds along TNTs [142]. The NMeds used were composed of the FDA-approved biocompatible and biodegradable polymer poly(L-lactic-co-glycolide) (PLGA), which was surface decorated with the g7 peptide [143,144], known to promote BBB crossing and CNS accumulation. These NMeds were administered to cultures of glial cells or to co-cultures of neuronal and glial cells. Remarkably, the authors were able to demonstrate both the homotypical transport of targeted NMeds between glial cells and also the heterotypical exchange from glial cells to neuronal cells. This piece of information holds great importance for therapies: often, for neurodegenerative pathologies, researchers aim to have selective targeting to neurons, which is difficult to achieve. Promoting the formation of TNTs could, therefore, represent a possibility to enhance the transport of NMeds from glial cells to neurons. To pursue this hypothesis, the authors also demonstrated a 2-fold increase in the number of TNTs formed by glial and neuronal cells after transfection with the protein mSEC, known to enhance the formation of TNTs. Interestingly, the transport of NMeds among cells increased by almost 25%, along with the increased number of TNTs. This study highlights how crucial it is to investigate how NMeds impact TNT formation in order to design ways to modulate their formation with transfection, other molecules that can be more easily administered with the NMeds, or even that could be co-encapsulated with the therapeutic pharmaco in the NMed formulation.

#### 3.2.2. Lipidic NMeds

Lipid-based NMeds are now on the cutting edge of nanomedicine development. This has been largely due to the recent global pandemic caused by SARS-CoV-2, for which the primary vaccine is a lipidic NMed [145]. With the increase in NMed use on the global level, it is important to carefully study how TNTs will come into play for the biodistribution and biological response of these treatments. The first study to analyze lipidic NMeds interacting with TNTs was by Kristl et al. [146]. In this interesting study, the authors administered solid lipid nanoparticles (SLNs) composed of compritol to cultured keratinocytes, revealing that the SLNs were actively transported along thick TNTs between cells. Notably, a comparison in the number of TNTs formed by SLN-treated and untreated keratinocytes was also performed. Experiments where the cells were treated with the SLNs showed an increased TNT formation rate compared to the controls. This highlights the stressful effect of the NMeds on cell cultures, but also the importance of studying how they interact with TNTs to decide their final fate and the significance of their biological effect. In fact, these data show that the presence of NMeds could directly affect the exchange of materials between cells even without any specific molecular trigger. 

Astanina et al. also investigated the impact of TNT modulation by fatty acids on the exchange of lipid droplets [147]. In this study, they tested the effect of arachidonic and stearic acid on the formation rate of TNTs in a primary culture of endothelial cells dosed with NMeds. The authors reported that no difference was observed after the administration of stearic acid, while arachidonic acid led to a 4-fold increase in the number of TNTs. This difference might lay in the role of arachidonic acid, which promotes migration and metabolic activity in the cells [148]. Remarkably, this increase in TNT formation led to a 3-fold increase in the exchange of NMeds compared to the control. These data highlight that the formation rate of TNTs is not linearly correlated with the transport of NMeds, although they are influenced by each other.

**Figure 3:**
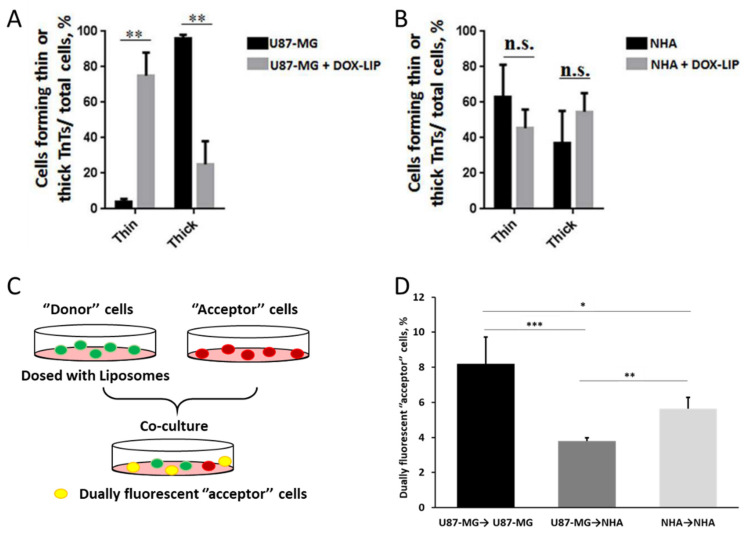
Type of TNTs in GBM and healthy astrocytes, and exchange of liposomes via TNTs. (**A**) Type of TNTs formed by GBM cells before and after administration of doxorubicin. (**B**) Type of TNTs formed by healthy astrocytes before and after administration of doxorubicin. Data are expressed as mean ± SE from three independent experiments. Data were analyzed by two-way ANOVA followed by Sidak’s multiple comparisons test; n.s., not significant; ** *p* < 0.01 (**C**) Experimental protocol to study TNT formation in co-cultures using different fluorophores to distinguish cell types. (**D**) Homotypical vs. heterotypical transfer via TNTs of doxorubicin-loaded liposomes in co-cultures of GBM and healthy astrocytes. U87-MG: GBM cells; NHA: normal human astrocytes; DOX: doxorubicin. *N* = 3 independent experiments. * *p* < 0.05; ** *p* < 0.01; *** *p* < 0.001 by Student *t*-test Reproduced with permission from Formicola et al. [149] (Frontiers in Bioengineering and Biotechnology; published by Frontiers; 2019).

A study by Formicola et al. recently highlighted that the type of TNTs formed by cells is also an important parameter to analyze [149]. In fact, “thick” TNTs are more efficient in the transport of material compared to “thin” ones. The authors here showed a difference in the composition of TNTs between two cell types: while GBM cells tended to form more stable “thick” TNTs, healthy astrocytes more frequently formed “thin” TNTs. Interestingly, the administration of free doxorubicin [150,151,152,153] induced a shift in this ratio between “thick” and “thin” TNTs for GBM cells, while astrocytes were unaffected. In particular, the majority of TNTs formed by GBM cells after the administration of the drug was of the “thin” type, similar to healthy astrocytes. Notably, the administration of doxorubicin-loaded liposomes produced the same effect on the composition of TNTs in both cell types (Figure 3A,B). This aspect needs to be properly investigated to assess the implications of this shift and to understand how to possibly control this phenomenon accordingly. Moreover, groundbreaking results presented in this study further underlined the importance of investigating the impact of targeting ligands. In this study, the authors decorated liposomes with ApoE and chlorotoxin, two moieties used for GBM targeting, and studied their trafficking via TNTs. In particular, they administered these NMeds to co-cultures of U87GM and human astrocytes cells (Figure 3C). Notably, the authors reported that targeted liposomes were actively transported via TNTs in co-cultures; however, a significant difference was seen in the direction of movement. In fact, homotypical transfer GBM→GBM and astrocyte→astrocytes was significantly more frequent compared to heterotypical transfer GBM→astrocyte (Figure 3D). These data demonstrate a pivotal point in the future design of NMeds for GBM treatment. This could be a good indication that the efficacy of targeted NMeds could be enhanced by the homotypic transfer of drugs between GBM cells while preserving the health of nearby healthy astrocytes. These various studies show the complexity of TNT research and their potential role in NMed therapeutics. On one side, this could be helpful to improve the spread of NMeds between localized cells, but on the other, it could be detrimental if the targeted cells spread the formulation to cells that were not the intended target. 

## 4. Limitations in Tunneling Nanotubes Detection

**Figure 4:**
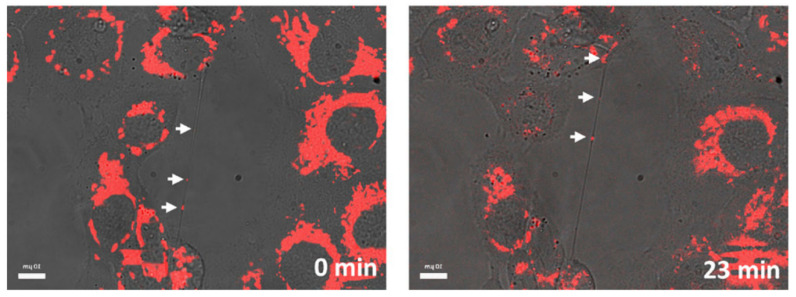
Representative imaging of TNTs using a combination of transmitted light and fluorescently labelled NMeds by confocal microscope. Reproduced with permission from Sáenz-de-Santa-María et al. [140] (Oncotarget; 2017).

As described in the previous chapters, TNTs have been detected in a plethora of models and have been shown to have a notable effect in NMed delivery. They have been found in immortalized or primary cell cultures [154], but also in spheroids [38,140] and organoids [155], as well as in vivo [33,156]; however, evidence of TNTs in tissues in vivo is less prominent when compared to the abundance of studies about TNTs in vitro. It is crucial, though, to highlight that the true number or rate of TNT formation in vivo is still probably underestimated. In fact, this possible misinterpretation of results could arise from the difficulty of detecting TNTs in samples, which is hampered by several problems in the imaging techniques available. These difficulties are predominantly linked to the fragile and transient structure of TNTs. These membranous tubules are often difficult to image, even in cultured cells in vitro, due to the numerous and arduous treatments necessary to prepare samples for imaging via electron, atomic force, or confocal microscopy, which can damage or destroy the projections. These problems linked to sample processing are exacerbated when animal tissue samples are involved, due to the fixation and preparation methods required. For these reasons, confocal microscopy is the preferred technique for visualizing TNTs due to the less arduous sample preparation while maintaining high resolution at the nanoscale via STED (Stimulated Emission Depletion) and spinning disk imaging [157]. While confocal microscopy offers many advantages and is the preferred method for imaging TNTs, another difficulty arises in the fact that there are few viable methods to specifically image TNTs. To clarify, TNTs are composed of cytoskeletal components and cell membranes. This means that any staining with antibodies for actin, tubulin, or plasma membranes will most likely result in high background fluorescence throughout the whole sample, with no distinction between different types of cell protrusions. Thus, one of the most used techniques to visualize TNTs both in vitro and in vivo is to combine a highly specific fluorescent staining for the object of interest combined with a transmitted light imaging. With this approach, it is possible to visualize the structure of the TNTs and the cargo transported along the tubules simultaneously (Figure 4). It is important to note that this method is mainly applicable to cell cultures where ultra-thin tissue sections of only a few µm are necessary to exploit the combination of fluorescence and light transmission images. This highlights the importance of investigating new techniques and protocols for TNT imaging, along with researching methods to tune their formation. In particular, a new antibody, specific for TNTs, would represent a huge improvement in TNT studies, with positive implications also for more complex samples from tissues. With all of the processing required, true in vivo experiments would ideally be able to track TNTs in real time, but up until now, this has not been achieved and is a critical next step to understand their biological relevance.

## 5. Conclusions and Future Prospects

Cells have been biologically programmed to share material, both to benefit from shared material such as proteins and organelles, and to dilute toxins. For this reason, cells naturally create connections such as TNTs to fulfill this need. While this can be a positive trait that allows for cell survival, they can represent a highway for the spreading of dangerous materials and even pathogens (viruses and bacteria), which take advantage of these connections to avoid the immune system. This opens up a Pandora’s box for researchers to use these pathways to deliver NMeds in a more controlled way, blocking or exploiting these connections. In fact, starting from the knowledge that NMeds can be transferred to other cells after uptake, it is of crucial significance to understand the dynamics that trigger TNT trafficking in order to take advantage of it. Hampering the formation of TNTs can help increase drug accumulation in the target cell while avoiding off-target toxicity; however, increasing these connections could multiply the therapeutic effect of delivered pharmaceutics throughout a tissue. Either way, TNTs need to be deeply investigated in their interaction with targeted NMeds: if the NMeds arrive at the targeted cells but are then transferred to other cells, the targeting effect is minimized. On the other hand, these connections could be used to enhance delivery between cells and promote drug delivery to difficult-to-reach cell populations.

In this work, we reviewed the work that has been completed in this direction, focusing on the core material of the NMeds. Overall, we found that both inorganic and organic NMeds are trafficked along TNTs, although differences in the rate of exchange were evidenced. Unfortunately, it is still unclear whether these differences are to be attributed to the NMeds or to the cell types, as it was demonstrated that different cells use TNTs with different rates. It would be, therefore, necessary to perform a more comprehensive investigation on the impact that different NMeds have on the same cell type, in terms of the number of TNTs formed, the extent of NMed exchange, and the type of TNTs (“thick” or “thin”). At the same time, literature that characterizes the effect of a single type of NMed on TNT formation in different cell types is lacking. Information about this will be crucial for researchers to better understand how NMeds, both targeted and untargeted, can be exchanged between cells, and to predict whether TNTs are promoting or reducing the therapeutic efficacy of NMeds.

In addition to the core material, there are several other parameters of NMeds that are to be considered when investigating their transport via TNTs, such as: size, hydrophilicity, surface engineering, stiffness, shape, surface charge, and the amount and type of drug loaded into the NMeds. Surface charge, for example, is one of the most important features for NMeds, as it can affect biodistribution, toxicity, and immunogenicity. It could be hypothesized that a positively charged NMed would trigger the formation of TNTs due to a higher toxicity compared to those that are negatively charged. This could potentially promote the spread of NMeds in the whole targeted tissue. Following the same rationale, the drug loaded into NMeds could also have a direct effect on the TNT formation rate. A higher amount of drug, both from higher loading content or faster release, could in fact increase the stress level of the targeted cell, thus promoting the formation of a higher number of TNTs and the spreading of the drug to other cells. At the same time, co-encapsulation of an inhibitor of TNT formation such as metformin could determine an accumulation of drug in the target cell and/or a reduction in the spreading of diseases.

The study of TNTs is still in its infancy, but results are already demonstrating the theoretical importance that they offer in NMed treatments. New methodology and further in-depth studies will be crucial to better understand and potentially control this currently un-utilized process of cell-to-cell transfer. Altogether, these data will be pivotal for giving researchers a clearer picture of how our technological tools, i.e., NMeds, can be optimized and specialized using TNTs.
